# Parasitic Helminths and Control Strategies

**DOI:** 10.3390/pathogens15101060

**Published:** 2026-10-08

**Authors:** Jackson Victor de Araújo, Luís Madeira de Carvalho, Fabio Ribeiro Braga

**Affiliations:** 1 Department of Veterinary Medicine, Federal University of Viçosa, Viçosa 36570-900, Brazil; 2 Programa de Pós-Graduação em Medicina Veterinária, Universidade Federal Fluminense, Avenida Almirante Ary Parreiras, 507, Niterói 24230-321, Brazil; 3 Department of Animal Health, Faculty of Veterinary Medicine, University of Lisbon, 1300-477 Lisboa, Portugal; madeiradecarvalho@fmv.ulisboa.pt; 4 Laboratório de Parasitologia Experimental e Controle Biológico, Universidade Vila Velha, Rua São João, 48, Vila Velha 29102-920, Brazil

This Special Issue brought together eleven contributions that illustrated, from different perspectives, the current state of research on parasitic helminths and the strategies available for their control, organized into four themes: (a) epidemiology and surveillance in public health, (b) the parasite–host relationship, (c) sustainable management in domestic and production animals, and (d) therapeutic innovation. (a) Epidemiology and surveillance in public health: Geohelminth–malaria co-infection in pregnant women in Kinshasa was investigated, with increased risk in the third trimester of pregnancy [1], and a systematic review compiled 248 studies on alveolar echinococcosis published between 2020 and 2025, showing diagnostic advances and persistent challenges in disease detection and treatment [2]. (b) The parasite–host relationship: Helminth antigens modulate SARS-CoV-2-induced T cell responses in individuals infected with *Onchocerca volvulus* [3]; metacercariae of *Cotylurus cornutu*s were identified in *Lithoglyphus naticoides*, with different infection rates between host sexes [4]; and sociability linked to reproductive status was associated with differences in intestinal infections of the red-billed chough [5]. (c) Sustainable management in domestic and production animals: The relationship between mean and variance of fecal egg counts in horses provided a practical basis for selective treatment decisions [6]; the application of *Mucor circinelloides* spores to the soil reduced the viability of *Ascaridia galli* and *Capillaria* spp. eggs in free-range systems [7]; formulation strategies for fungal biocontrol, including sodium alginate pellets containing *Duddingtonia flagrans*, were reviewed in detail [8]; and the evidence on sheep genetic resistance to gastrointestinal nematodes was synthesized, highlighting candidate biomarkers for genomic selection [9]. (d) Therapeutic innovation: A safe and effective oral treatment was reported for lungworm infections in hedgehogs in Greece [10], and medicinal plants, phytochemicals, and inorganic nanoparticles with activity against *Strongyloides stercoralis* were reviewed as emerging alternatives [11]. Together, these contributions reflect the diversity of approaches used in helminth control, from surveillance and epidemiology to molecular biology, ecology, biocontrol, and natural products, under the One Health perspective.

For decades, the control of gastrointestinal helminthiasis in public health has relied almost exclusively on anthelmintics. However, times have changed, raising the question: have we made progress or regressed in helminth control?

Several factors now influence decision-making in this field, which is closely linked to One Health. The main ones are anthelmintic resistance (AR) in livestock herds [12], already recognized as a serious challenge; climate change, which may alter parasite epidemiology in different regions of the world; and the demand for more sustainable control models. These challenges point toward a broader research agenda, in which alternative control strategies, biotechnology, and omics technologies support the development of more targeted approaches based on molecular mechanisms [8,13,14,15]. In human health, the control of helminth infections aims mainly at alleviating suffering, reducing poverty, and promoting equal opportunities between men and women [16]. For decades, the scientific community has warned that the history of anthelmintic resistance in veterinary medicine could help us understand potential resistance in human helminths [17,18]. Ng’etich et al. [19] noted that anthelmintic resistance in humans is an emerging challenge; integrated strategies should therefore follow One Health principles, combining the rational use of medicines, surveillance, and environmental interventions. In this regard, and returning to the initial question, the future of helminth control should not depend on the search for a new anthelmintic, but rather on integration across diagnosis, knowledge of parasite epidemiology, sustainable management, biological control, biotechnology, and targeted approaches aligned with the One Health framework [12,19].

Parasitic diseases in domestic animals remain a major challenge to animal, human, and environmental health, and require more effective, sustainable, and integrated control strategies [20]. A paradigm shift is needed, with emphasis on sustainable management. Traditional deworming programs, once considered fully effective, are no longer compatible with the current scenario of high anthelmintic resistance or with the production pressures of livestock farming. There is a clear need for evidence-based control, coproparasitological monitoring, epidemiological knowledge of parasitic diseases, strategies tailored to each production system, and animal health surveillance [21]. Within this scenario, two contributions of this Special Issue illustrate how management and genetics support this transition: selective treatment decisions in horses benefit from the practical use of mean fecal egg counts and their variability [6], and genetic selection programs in sheep rely on candidate biomarkers identified through omics approaches [9].

In this editorial, we suggest that anthelmintic resistance in animals, humans, and the environment should be addressed within the One Health context, given how closely these domains are linked. AR is no longer a local problem; it is now recognized as a global issue [22]. Vercruysse et al. [18] pointed out that, although there was no evidence of widespread resistance in human helminths at the time, the reduced efficacy of benzimidazoles against *Trichuris trichiura* already justified surveillance systems to continuously monitor anthelmintic performance. Diawara et al. [23] identified an increase in the frequency of β-tubulin gene polymorphisms in geohelminth populations repeatedly exposed to albendazole, suggesting that the selective pressure of preventive chemotherapy may favor alleles associated with AR. Ng’etich et al. [19] proposed that anthelmintic use in humans and animals, together with environmental contamination by drug residues and infective forms, forms an interdependent system for the selection and spread of resistance genes. Within this scenario, the review by Sinhalage et al. [9] in this Special Issue shows how transcriptomics and other omics approaches can identify candidate biomarkers to monitor and support resistance to gastrointestinal nematodes in sheep.

The use of new technologies against gastrointestinal helminthiasis has received growing attention in recent years, particularly biological control. The first report on this topic dates back to the 1970s, when the in vitro activity of biological control agents against human helminth eggs was proposed [24]; at that time, the One Health concept was not yet widely accepted. That study showed biological control of the geohelminth *Ascaris lumbricoides*, an innovation for its period. Following this line of research, Araujo et al. [25] showed that nematophagous fungi, acting as biological control agents, were effective under laboratory conditions in destroying the eggs of the zoonotic nematode *Toxocara canis*. Later, Braga et al. [26] resumed research on the biological control of *Ascaris lumbricoides* eggs and found that sustainable environmental management could act as a synergistic tool for controlling ascariasis. Nearly 50 years after the first report on *A. lumbricoides* eggs, research has advanced considerably, and biotechnology is now widely applied in this field, from the study of metabolites of biocontrol fungi to the production of silver nanoparticles. Ferraz et al. [15] documented the effect of silver nanoparticles on *A. lumbricoides* eggs, a technology derived from the nematophagous fungus *Duddingtonia flagrans* with potential application against the environmental stages of this parasite. Formulation strategies for fungal biocontrol agents, including pelletized formulations of *D. flagrans*, are currently reviewed in detail in this Special Issue [8].

Ng’etich et al. [19] argued that sustainable control depends on the integration of human health, animal health, and the environment, addressing the rational use of anthelmintics, surveillance of anthelmintic resistance, basic sanitation, and health education. Geohelminthiasis is a clear example of this interface, and joint efforts across different areas of expertise are therefore important. Ellwanger and Cavallero [27] further emphasized that environmental degradation and socioeconomic inequalities also contribute to the conditions that favor geohelminth transmission. The efforts of the World Health Organization’s Department of Neglected Tropical Diseases (WHO/NTD) deserve attention here. The department published an important document complementing its main strategy, the Road Map for Neglected Tropical Diseases 2021–2030, which raises awareness of the need for continued mobilization and investment to reduce the burden of disease for millions of people worldwide [28]. This document establishes integrated surveillance within the One Health framework, with priorities for the sustainable control of helminthiasis. In line with this agenda, two contributions of this Special Issue address public health surveillance directly: geohelminth–malaria co-infection in pregnant women in Kinshasa [1] and a systematic review of alveolar echinococcosis in the early 2020s [2].

The future of helminth control will depend on our ability to integrate advances in molecular diagnostics, omics technologies, biological control, and the One Health approach into practical and sustainable strategies. Rather than replacing conventional approaches, these tools should complement current control programs and help address challenges such as anthelmintic resistance, environmental contamination, and the complexity of parasite transmission. Research efforts are currently focused on identifying molecular markers of resistance and improving methods for monitoring treatment efficacy. This Special Issue brought together eleven contributions reflecting the diversity of helminthology research, from epidemiological studies and parasite–host interactions to molecular characterization, omics technologies, biological control, and natural products. A second volume is planned to continue this series.

## References

[1] Dianzenza C., Tshiongo J.K., Kuseke L., Muswele F., Kasereka G., Mangungulu D., Laken E.K., Tevuzula V.M., Kayentao K., Mavoko H.M. (2026). Geohelminthiasis and Malaria Co-Infection in Pregnant Women in Kinshasa: Case of Lisungi and Lukunga Hospitals in Democratic Republic of the Congo. Pathogens.

[2] Ciomaga B.-F., Hogea M.-O., Muntean A.-A., Muntean M.-M., Popa M.I., Popa G.L. (2026). Alveolar Echinococcosis in the Early 2020s: A Systematic Review. Pathogens.

[3] Nembot Fogang B.A., Arndts K., Adjobimey T., Owusu M., Opoku V.S., Adu Mensah D., Boateng J., Osei-Mensah J., Meyer J., Klarmann-Schulz U. (2026). Helminth Antigens Modulate Virus-Induced Activation of CD154 (CD40L) Expression on T Cells in *Onchocerca volvulus*-Infected Individuals. Pathogens.

[4] Stanevičiūtė G., Stunžėnas V., Petkevičiūtė R. (2025). Morphological and Molecular Studies of Tetracotyle-Type Metacercariae of the Genus *Cotylurus* Szidat, 1928 (Trematoda) from the Gravel Snail *Lithoglyphus naticoides* (Gastropoda) and Host Sex Dependent Differences in Infection Rate. Pathogens.

[5] Blanco G., Garijo-Toledo M.M., González del Barrio J.L., Frías Ó., Montoya Ayala R., Palacios-Martínez I. (2025). Sociability Linked to Reproductive Status Affects Intestinal Parasite Infections in the Red-Billed Chough. Pathogens.

[6] Cabaret J., Guerrero Molina C., Martínez-Ortiz-de-Montellano C., Alcala Canto Y. (2026). Relationship Between Mean Faecal Gastrointestinal Nematode Egg Excretion in Horses and Its Variability: Implications for Control. Pathogens.

[7] León J.A., Pérez-Anzúrez G., Abreu Ramos I., Magos Amado C.E., Boso Dafonte D., Lozano J., Hernández Malagón J.Á., Cazapal-Monteiro C., Bonilla R., Sanchís J. (2025). Testing a Sustainable Strategy Against Poultry Helminth Stages Developing in the Soil. Pathogens.

[8] Fonseca J.D.S., do Carmo T.A., Vital B.D.O.B., Monteiro T.S.A., Balbino H.M., Santos H.A., Tebaldi de Queiroz V., Braga F.R., de Araújo J.V. (2026). Formulation Strategies for Fungal Biocontrol of Gastrointestinal Helminths in Domestic Animals and Plant-Parasitic Nematodes: A Review. Pathogens.

[9] Sinhalage K., Polizel G.H.G., Karrow N.A., Schenkel F.S., Cánovas Á. (2026). Sheep Genetic Resistance to Gastrointestinal Nematode Infections: Current Insights from Transcriptomics and Other OMICs Technologies—A Review. Pathogens.

[10] Markakis G., Symeonidou I., Komnenou A., Beugnet F., Ganoti M., Papadopoulos E. (2026). Prevalence of Lungworm Infections in Hedgehogs (*Erinaceus roumanicus*) in Greece and a Novel Therapeutic Approach for Treatment. Pathogens.

[11] López-Abán J., Vicente-Santiago B., Gutiérrez-Soto G., Rodríguez-Garza N.E., Kačániová M., López-Sandin I., Romo-Sáenz C.I., Ballesteros-Torres J.M., Galaviz-Silva L., Castillo-Velázquez U. (2025). Emerging Approaches to Anthelmintic Therapy Using Medicinal Plants and Phytochemicals: A Review of Natural Products Against Strongyloidiasis. Pathogens.

[12] Kaplan R.M. (2004). Drug resistance in nematodes of veterinary importance: A status report. Trends Parasitol..

[13] McVeigh P. (2020). Post-genomic progress in helminth parasitology. Parasitology.

[14] Mendoza-de Gives P., Braga F.R., Araújo J.V. (2022). Nematophagous fungi, an extraordinary tool for controlling ruminant parasitic nematodes and other biotechnological applications. Biocontrol Sci. Technol..

[15] Ferraz C.M., Assis J.P.B., Guerini E.C., Leal J.V., Soares F.E.F., Fronza M., de Araujo J.V., Carvalho L.M., Braga F.R. (2026). Ultrastructural Evaluation of *Ascaris lumbricoides* Eggs Treated with Silver Nanoparticles Biosynthesised by *Duddingtonia flagrans* Using Scanning Electron Microscopy. Pathogens.

[16] World Health Organization (2006). Preventive Chemotherapy in Human Helminthiasis: Coordinated Use of Anthelminthic Drugs in Control Interventions: A Manual for Health Professionals and Programme Managers.

[17] Geerts S., Gryseels B. (2000). Drug resistance in human helminths: Current situation and lessons from livestock. Clin. Microbiol. Rev..

[18] Vercruysse J., Albonico M., Behnke J.M., Kotze A.C., Prichard R.K., McCarthy J.S., Montresor A., Levecke B. (2011). Is anthelmintic resistance a concern for the control of human soil-transmitted helminths?. Int. J. Parasitol. Drugs Drug Resist..

[19] Ng’etich A.I., Amoah I.D., Bux F., Kumari S. (2024). Anthelmintic resistance in soil-transmitted helminths: One-Health considerations. Parasitol. Res..

[20] Uilenberg G. (1996). Integrated control of tropical animal parasitoses. Trop. Anim. Health Prod..

[21] Kaplan R.M., Nielsen M.K. (2010). An evidence-based approach to equine parasite control: It ain’t the 60s anymore. Equine Vet. Educ..

[22] Kaplan R.M., Vidyashankar A.N. (2012). An inconvenient truth: Global worming and anthelmintic resistance. Vet. Parasitol..

[23] Diawara A., Halpenny C.M., Churcher T.S., Mwandawiro C., Kihara J., Kaplan R.M., Streit T.G., Idaghdour Y., Scott M.E., Basáñez M.G. (2013). Association between response to albendazole treatment and β-tubulin genotype frequencies in soil-transmitted helminths. PLoS Negl. Trop. Dis..

[24] Lysek H. (1976). Classification of ovicide fungi according to type of ovicidity. Acta Univ. Palacki. Olomuc..

[25] Araújo J.V., Santos M.A., Ferraz S. (1995). Efeito ovicida de fungos nematófagos sobre ovos embrionados de *Toxocara canis*. Arq. Bras. Med. Vet. Zootec..

[26] Braga F.R., Araújo J.V., Campos A.K., Carvalho R.O., Silva A.R., Tavela A.O., Maciel A.S. (2007). Observação in vitro da ação dos isolados fúngicos Duddingtonia flagrans, Monacrosporium thaumasium e Verticillium chlamydosporium sobre ovos de Ascaris lumbricoides (Lineu, 1758). Rev. Soc. Bras. Med. Trop..

[27] Ellwanger J.H., Cavallero S. (2023). Editorial: Soil-transmitted helminth infections from a One Health perspective. Front. Med..

[28] World Health Organization (2022). Ending the Neglect to Attain the Sustainable Development Goals: A Rationale for Continued Investment in Tackling Neglected Tropical Diseases 2021–2030.

